# Supplementary material for: Crosstalk between SHH and FGFR Signaling Pathways Controls Tissue Invasion in Medulloblastoma
Source: Cancers (Basel). 2019 Dec 10;11(12):1985. doi: 10.3390/cancers11121985 (PMC6966681; doi:10.3390/cancers11121985)

Supplementary figures

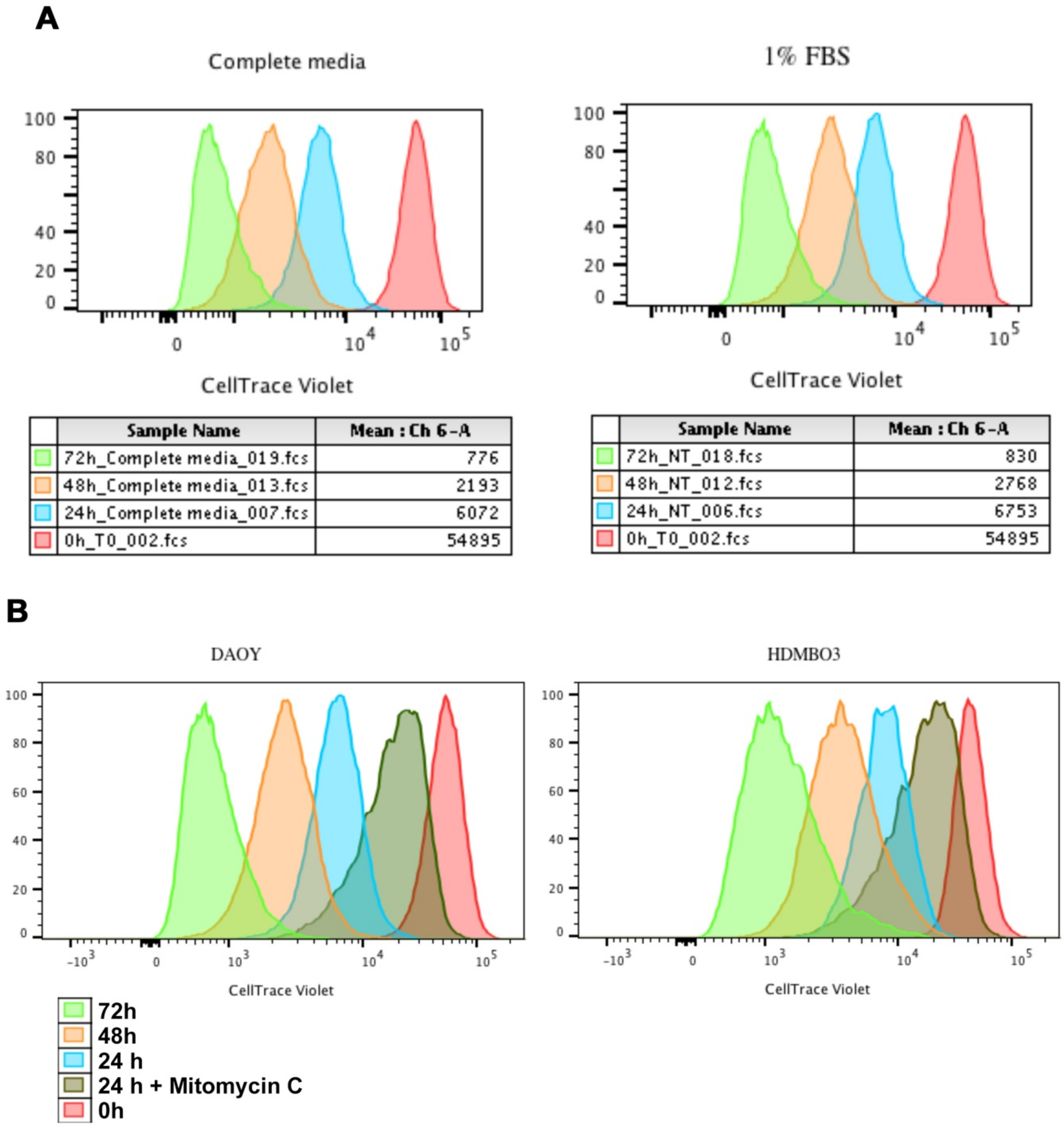

Figure S1: Validation of CellTrace violet quantification of proliferation A) Flow cytometry analysis of CellTrace violet stained cells in complete (10% FBS) or low FBS medium. B) Time course of dye dilution and confirmation of dye retention in DAOY and HD-MBO3 cells after mitotic blockade by mitomycin C treatment.

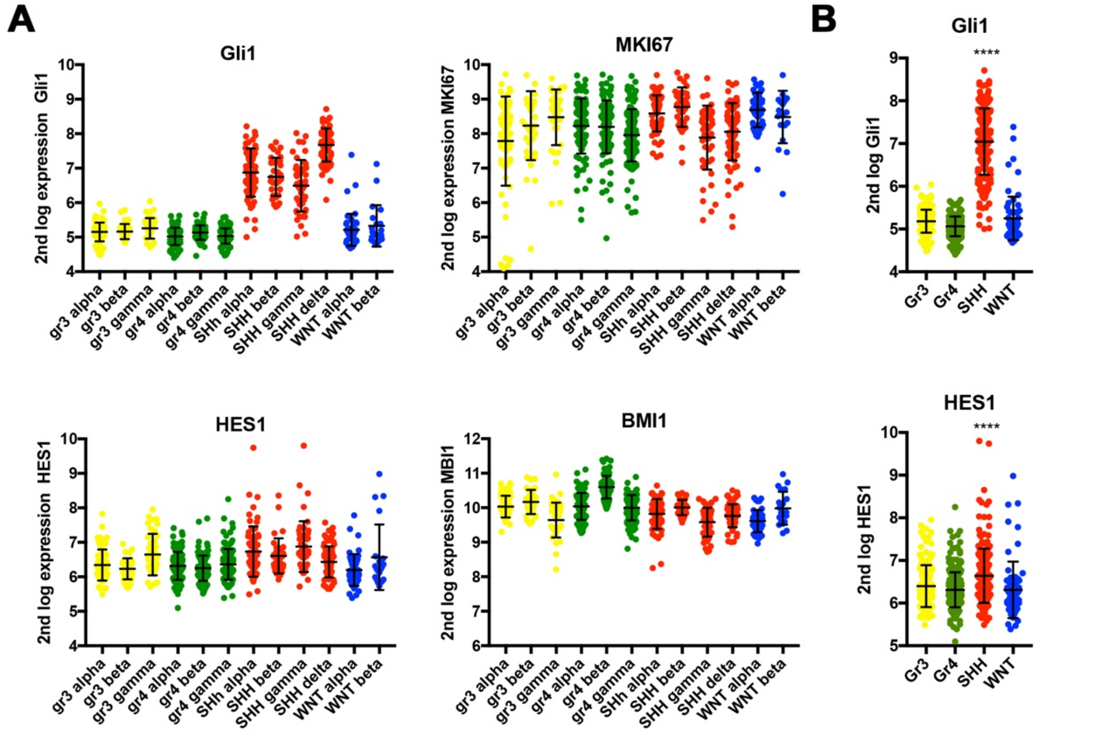

Figure S2: Gene expression analysis in primary tumors R2 database analysis of **(A)** subtype- or **(B)** subgroup-specific *Gli1*, *MKI67*, *HES1* and *BMI* expression in the Cavalli 763 dataset. Gene expression levels are expressed 2nd log.

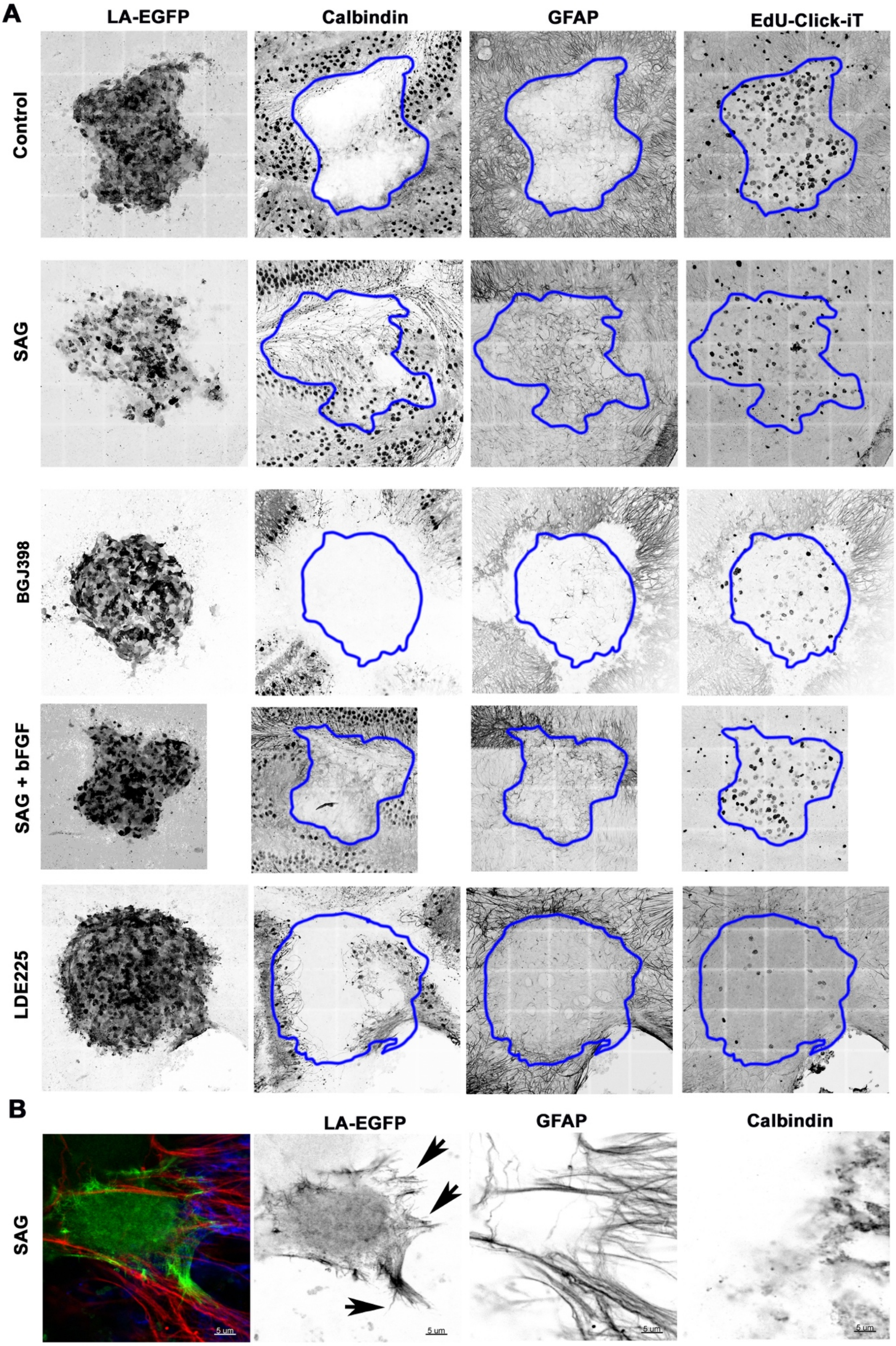

Figure S3: EdU staining in tumor cells implanted in OCSCs A) Confocal microscopy analysis of

spheroids in OCSCs five days after implantation with the treatments indicated. Blue lines indicate circumference of spheroids overlaid with the different staining in greyscale. **B)** High magnification confocal microscopy image of single invading cells after SAG treatment. Note invasive protrusion in LA-EGFP channel highlight with arrows.

Blots:

blots Fig. 1C

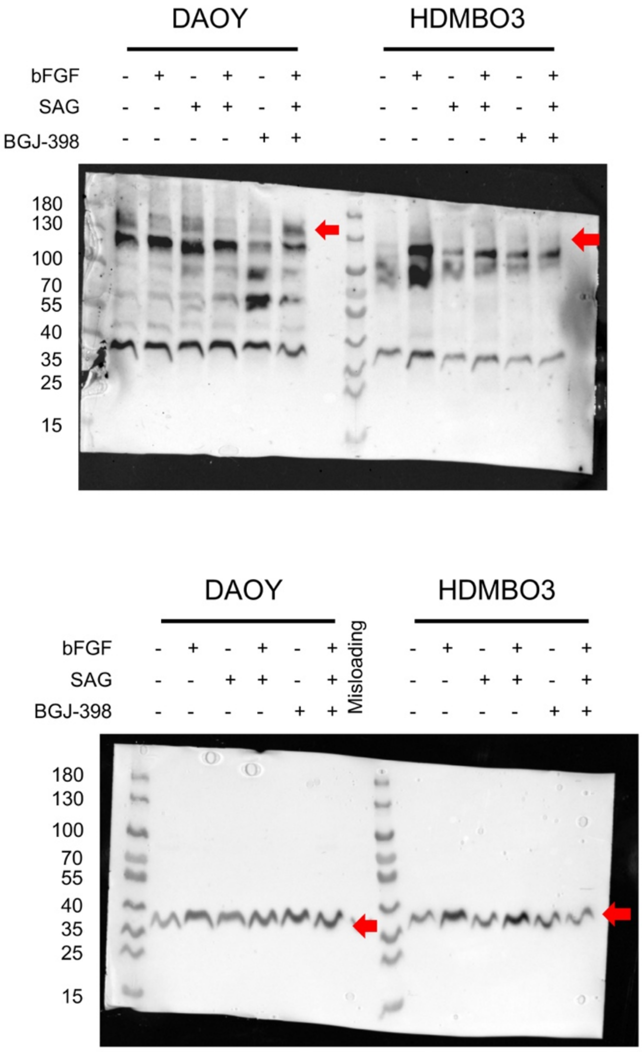

blots Fig. 1E

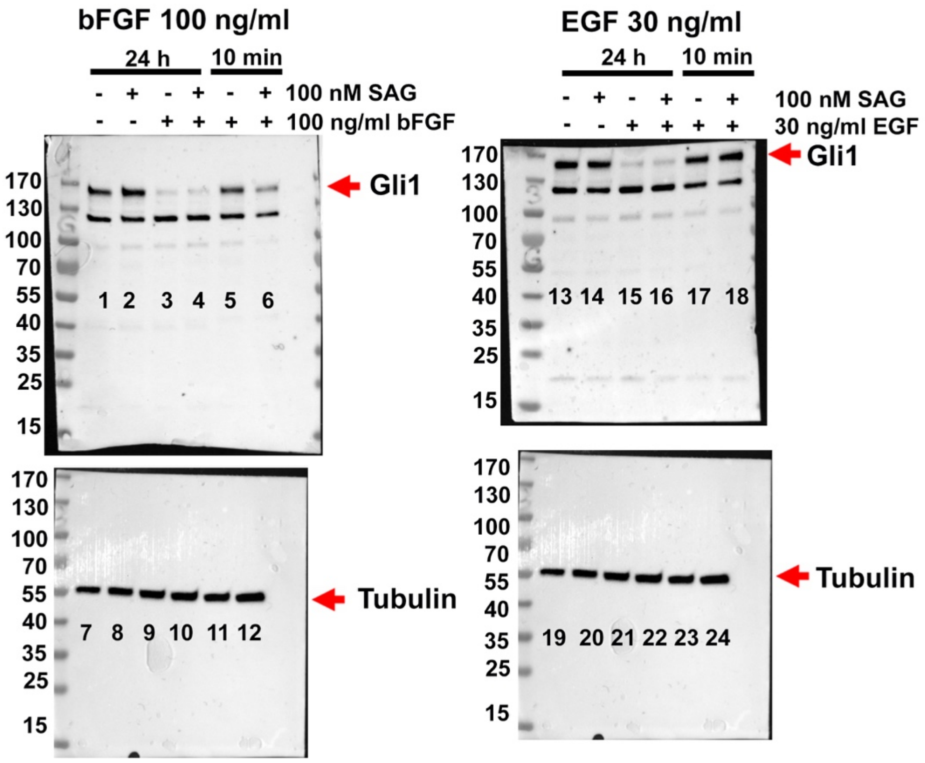

Gli1: 140 kDa im immunoblot

Tubulin: 55 kDa

blot Figure 4A

Fig. 4A, bFGF

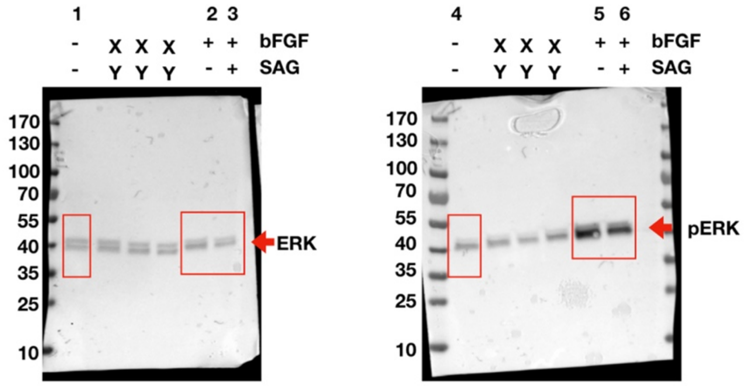

ERK 42-44 kDA

Fig. 4A, HGF

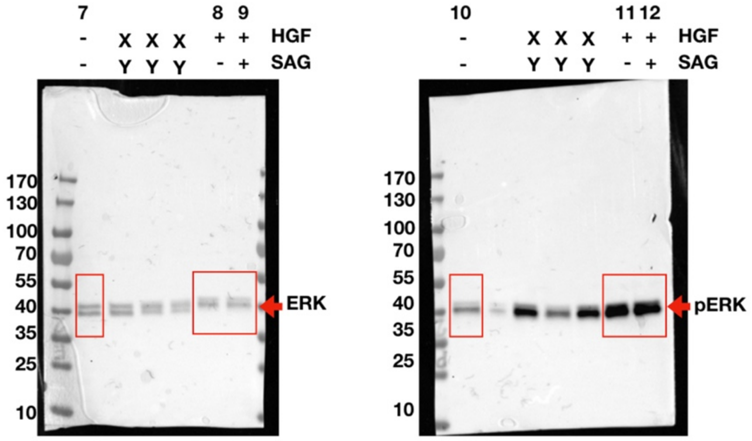

ERK 42-44 kDA

Fig. 4A, EGF

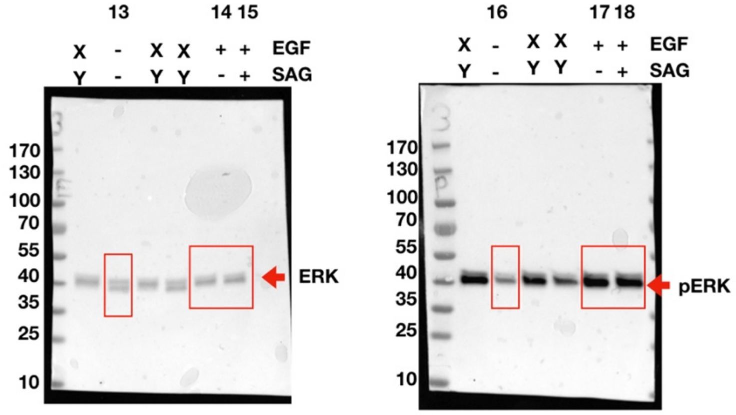

ERK 42-44 kDA

Figure 4B, left

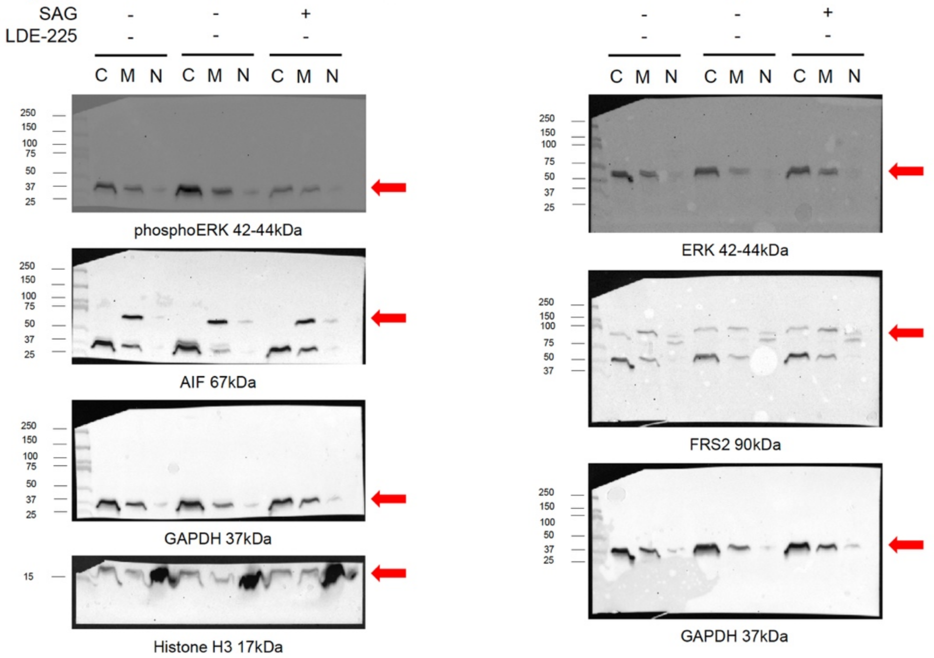

Figure 4B, right

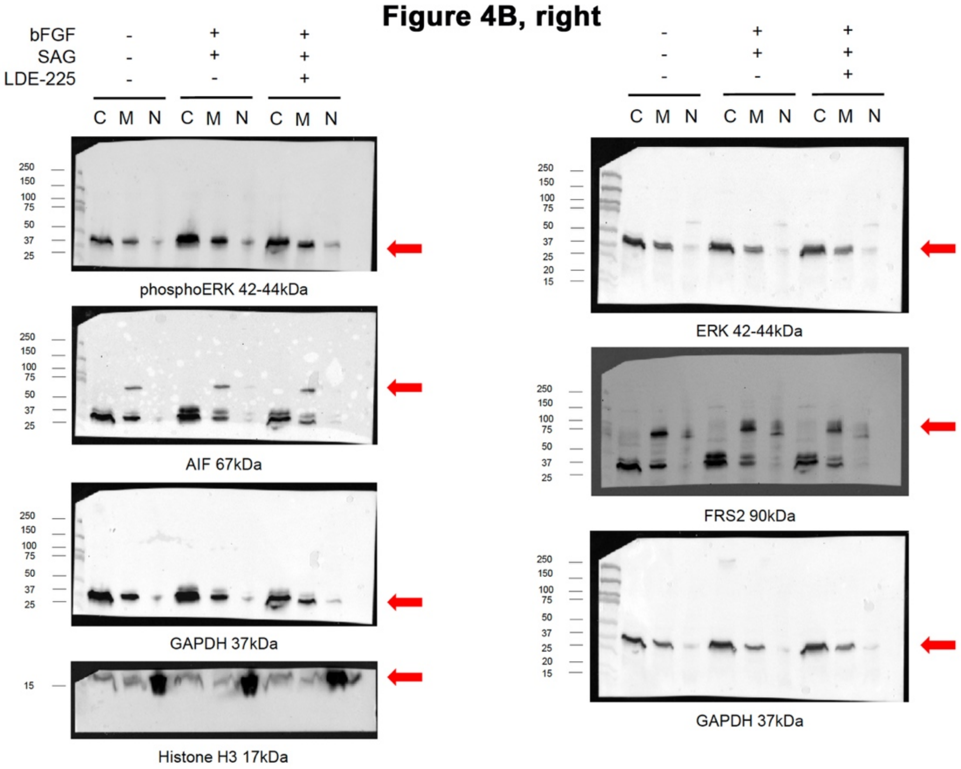

Supplement: Supplementary file 1 [file cancers-11-01985-s001.zip › Suppl-final/cancers-644016-suppl-final.pdf]
